# Problems associated with mass border testing of COVID-19

**DOI:** 10.1177/14034948211023659

**Published:** 2021-06-19

**Authors:** Mathias Grunér, Marika Nordberg, Knut Lönnroth

**Affiliations:** 1 Medimar Medical Clinic, Finland; 2 Åland Central Hospital, Finland; 3 Government of Åland, Finland; 4 Department of Global Public Health, Karolinska Institutet, Sweden

**Keywords:** SARS-Cov-2, COVID-19, screening, border restrictions, predictive values

## Abstract

We evaluated the yield of exit screening for SARS-Cov-2 performed in order for travellers to meet entry requirements to Sweden. Among 472 people screened, no infectious case of COVID-19 was detected, while two previously known cases were redetected after having already completed isolation. Our data suggest that depending on the epidemiological situation in the area of departure, border screening can lead to very low positive predictive values and very high costs per relevant case detected.

## Introduction

All Nordic countries have introduced various SARS-Cov-2 screening strategies and related entry restrictions for people crossing borders, although the empirical evidence for epidemiological impact of such measures remains limited [1]. The European Centre for Disease Prevention and Control has suggested the principles and rationale for border screening while stressing the need for continuous monitoring and evaluation [2].

On 6 February 2021, the Swedish government enacted a regulation that included a requirement for most non-Swedish residents >17 years of age to show a negative test certificate for SARS-Cov-2 as a condition to enter the country [3]. The test should be taken a maximum of 48 hours before arrival. Approved tests include polymerase chain reaction (PCR) tests and antigen tests. There is no exemption for people who have previously been infected with COVID-19, those with positive SARS-Cov-2 serology or vaccination against SARS-Cov-2 [4].

Åland is a Swedish-speaking self-governed island region of Finland with a population of about 30,000. Many people travel frequently to and from both the Finnish mainland and Sweden for work or study, to visit family, for tourism and other reasons. Although travel volumes have been reduced dramatically during the COVID-19 pandemic, necessary travel has continued.

The aims of this study were to determine the number and proportion of infectious COVID-19 cases detected through pre-departure PCR screening on Åland for the purpose of meeting entry requirements to Sweden and to validate whether an antigen test could potentially be a less costly alternative to the PCR test.

## Methods

We analysed the results of all pre-departure screening for SARS-Cov-2 in apparently healthy individuals done on Åland for the purpose of a test certificate from 8 February to 19 March 2021. The test certificates were almost exclusively needed for travel to Sweden. As the end destination or possible transit country, Sweden was the only direct international travel destination from Åland during the study period. The number of tests and test results were ascertained through a retrospective record review approved by the Åland Research Ethics Review Board (1/2020:5). This testing was centralised to one private health centre clinic with an on-site laboratory. Only PCR tests were used (real-time reverse transcriptase PCR, Mikrogen ampliCube Coronavirus SARS-CoV-2) with test sensitivity of 97% and specificity of 100% [5]. SARS-CoV-2 testing for other purposes was done at the outpatient infectious disease clinic at Åland Central Hospital. Any patient showing or describing any symptom of COVID-19 was referred to Åland Central Hospital following the applicable local guidelines. In parallel, verification of an antigen test (BIOSENSOR Standard Q COVID-19 Ag rapid test) was done using a separate validation sample of 28 known positive and 28 known negative PCR tests.

## Results

**Figure 1.**
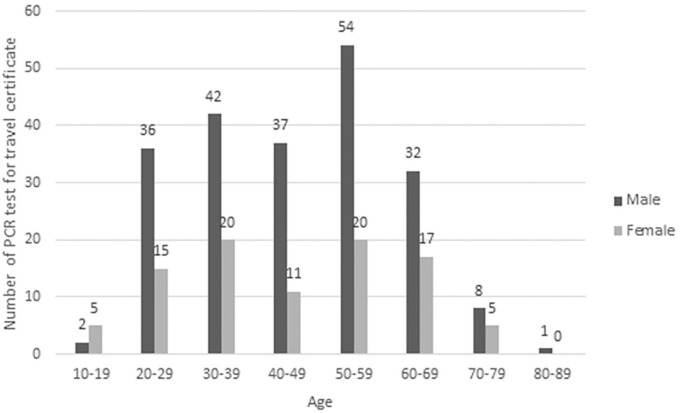
Age and sex distribution of tested individuals.

A total of 472 people were pre-departure tested, of whom 470 were negative. No one was referred due to symptoms. The age and sex distribution of tested individuals are shown in Figure 1. Two people (0.42%, 95% confidence interval (CI) 0.05–1.52%) tested positive, with Ct values of 33.03 and 37.12. Both had previously PCR-confirmed COVID-19 on Åland and had already been allowed to exit isolation before pre-departure testing. Excluding the two with previous infection, the one-sided 97.5% CI for the zero prevalence of infectious COVID-19 among the remaining 470 individuals was 0.00–0.78%.

The sensitivity of the antigen test was 60.7% (95% CI 40.6–78.5) and the specificity was 100% (95% CI 87.7–100). Borderline results were judged as negatives. Antigen detection in relation to PCR Ct value showed a negative result in all samples with Ct values >25.

## Discussion

**Figure 2.**
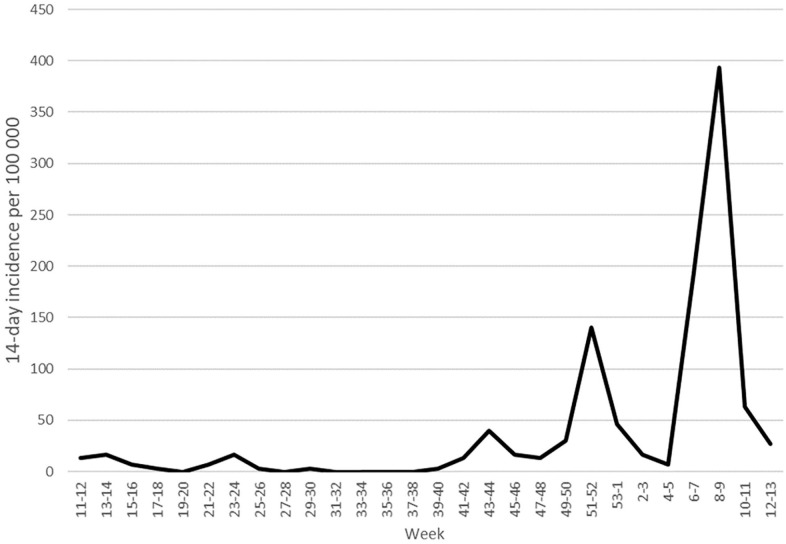
Fourteen-day incidence per 100,000 on Åland from 9 March 2020 to 4 April 2021. The test results reported here were from weeks 6–11 2021.

There was no meaningful yield of this screening, which was carried out during a rapidly shifting epidemiological period on Åland (Figure 2). At the beginning of the period of testing, Åland had had a four-week period with a 14-day COVID-19 incidence of <20/100,000. Starting on 16 February, Åland experienced the largest COVID-19 outbreak so far, during which the 14-day incidence reached >400/100,000 and then declined again to about 60/100,000. Assuming an average COVID-19 duration of 14 days, prevalence equals 14-day incidence. The maximum COVID-19 prevalence on Åland in this period would then be about 0.4%, which is within the CI for the test positivity. The number needed to test to detect one true case with this prevalence is 250. When prevalence is 0.02%, the number needed to test is 5000. The cost of a PCR test plus a certificate is about €250 Euro on Åland. This means that it would cost €62,500 to detect one true case when the prevalence is 0.4% and €1.25 million when the prevalence is 0.02%.

We re-detected two previously known cases. No isolation was imposed upon them, since they were previously known to the local health authorities. The positive test results were judged to be due to non-viable SARS-CoV-2 virus remnants, which can be detected for up to 12 weeks after symptom onset [6,7]. This finding underscores the importance of excluding people with known recent COVID-19 infection from test requirements. High test specificity and good test and analytical practice are not enough to ensure diagnostic accuracy. The interpretation of results in relation to clinical history and information about possible exposure is equally important. It is thus important to distinguish test accuracy from diagnostic accuracy. This becomes challenging during mass screening, and even a complete individual assessment cannot always discern whether a test is a true or false positive or negative, especially in an apparently healthy individual.

To reduce the overall cost for travel certificates. an antigen test was validated as an alternative to PCR. We found a sensitivity of 61%, which is in line with the pooled antigen test sensitivity for asymptomatic participants of 58.1% (95% CI 40.2–74.1%) reported in a recent Cochrane review [8]. Using an antigen test with lower sensitivity than PCR would reduce the risk of detecting irrelevant virus remnants after infection but also have a higher risk of missing infectious cases that can transmit to others.

Our data highlight three general problems with mass border testing, the importance of which depend on the epidemiological situation. The first of these problems is the potentially very low positive predictive values, especially when using a sensitivity PCR test in a moderate- to low-prevalence setting. The implication is that many would be unnecessarily prohibited from travel, and they may also be unnecessarily isolated and subjected to contact investigation unless proper assessment and cross-checking with local notification data are carried out. This problem diminishes with increasing prevalence. The second problem is that is potentially very low negative predictive values, especially when using an antigen test in a high-prevalence setting. The implication is that many infectious people may still be allowed to travel and may also get a false sense of security. Third, cost-effectiveness can be very low. The latter point seems not to be an issue for the country of arrival when the full cost is born by a foreign traveller. This may be judged appropriate if the travel is for pleasure, but it should at least be considered in border regions that are dependent on minimal border restrictions for sustainable social and economic development. The Nordic Council of Ministers, for example, has declared in its sustainable development plan that the Nordic countries should be ‘the world’s most integrated region’ [9].

High costs and a high proportion of false-positive findings during screening may be acceptable if the positive impact of such screening is clear. However, a recently updated Cochrane review concluded that there is a lack of ‘real-world’ evidence for travel-related control measures to contain the COVID-19 pandemic, including screening, and that the certainty of the evidence is low to very low [1]. Most studies on relevant epidemiological outcomes to date have been modelling studies, which is problematic when empirical evidence is very weak. Few studies have addressed the issue of false-positive results, especially those resulting from testing people with recent infection who carry virus remnants but are no longer infectious.
